# ASSESSMENT OF NATURAL RADIOACTIVITY LEVELS AND RADIATION EXPOSURE IN NEW BUILDING MATERIALS IN SPAIN

**DOI:** 10.1093/rpd/ncab089

**Published:** 2021-07-05

**Authors:** J L Mas, J R Caro Ramírez, S Hurtado Bermúdez, C Leiva Fernández

**Affiliations:** Dpto. Física Aplicada I, ETSI-Informática, Universidad de Sevilla, Avda. Reina Mercedes, s/n, Sevilla 41012, Spain; Mina Cobre Las Cruces, carretera SE-3410, km 4100.41860 Gerena, Sevilla, Spain; Dpto. Física Aplicada II, ETSA, Universidad de Sevilla, Avda. Reina Mercedes 2, Sevilla 41012, Spain; Dpto. Ingeniería Química y Ambiental, ETS-Ingeniería, Univ. Sevilla, C/de los Descubrimientos, s/n, Pabellón Plaza de América, Sevilla 41092, Spain

## Abstract

Novel building materials were manufactured and analyzed for ^226^Ra, ^232^Th and ^40^K using an HPGe gamma-ray spectrometer. The results show that the highest value of ^40^K was 4530 Bq per kg which was measured in a sample containing fly ashes from olive stones. The highest values of ^226^Ra and ^232^Th activities were 181 and 185 Bq per kg, which were measured in a sample with fly ashes from the co-combustion of coal and coke, respectively. On the other hand, the lowest values of ^40^K, ^226^Ra and ^232^Th activities were obtained for samples incorporating mussel shells. The radiological health hazard parameters, such as radium equivalent activity (Ra_eq_), activity concentration index (*I*), absorbed and effective dose rates, associated with these radionuclides were evaluated. These values are within the EU recommended limits in building materials, except for samples of concrete containing fly ashes from olive stones, coal and coke. This study has contributed to the inclusion of industrial wastes that have not been collected previously in the Naturally Occurring Radioactive Material (NORM) databases on radioactivity of building materials.

## INTRODUCTION

Natural radionuclides, such as ^40^K, ^238^U, ^232^Th and their respective progenies, are widely present in rock and soils, which are subsequently used to produce building materials. Their contribution to radiological hazard comes from external radiation and from the inhalation and uptake of radon and radon progeny. Worldwide mean activity concentrations in soil are 412 Bq per kg for ^40^K, 33 Bq per kg for ^238^U, 32 Bq per kg for ^226^Ra and 45 Bq per kg for ^232^Th^(1)^. It is worthy to mention that the corresponding activity concentration in worldwide average building materials is fairly similar: 500 Bq per kg (^40^K) and 50 Bq per kg (^226^Ra and ^232^Th)^(2)^. As a consequence, the worldwide average indoor external dose rate due to gamma emitters in building materials is relatively low, in the range of 84 nGy per h^(1)^.

Over the past years, special attention has been paid to save and preserves natural, nonrenewable materials at the European level. Waste material management is steered by the EU Waste Framework Directive (2008/98/EC)^(3)^, which sets a target at a minimum of 70% for the recycling of waste material. Therefore, scientific researchers have been focusing on finding new methods to produce environmentally friendly building materials. Most of these studies are based on the incorporation of wastes from different sectors (biomass, power plants, construction and demolition process) in building materials^(4–9)^. However, naturally occurring radioactive materials (NORM) are present in the products, by-products, residues and wastes materials.^(10)^. International radiation protection safety standards set the principles of protection concerning natural radioactivity, including NORM materials, establishing dose criteria for the maximum annual dose to control the radiation exposure to workers and members of the public^(11)^.

On the other hand, there was an increased focus on the identification of new alternatives to natural aggregates with the intention of conserving the natural aggregates and to maintain ecological balance. Several industries produce large volumes of waste representing a disposal and potentially environmental pollution problem, such as shells from the aquaculture industry or ashes of biomass combustion from the olive oil industry. The produced building materials incorporating those wastes have to be included in the database gathering radiological data on NORMs and construction materials^(12, 13)^. One of the main results drawn from the NORM databases was that just 41.7% of wastes produced an excess of gamma dose rate that was less than the recommended values (1 mSv per year), while this proportion raised up to 84.4% in the case of building materials^(13)^.

The aim of this work is to determine the activity concentrations of ^226^Ra, ^232^Th and ^40^K as well as the radiological health hazards associated with new building materials incorporating different bio-wastes (mussel shells and ashes from combustion of olive stones) from Spain. This study will also compare the obtained values with the building materials composed by using other industrial wastes. In addition, mollusk shells may act as a diluent of the radiological hazard of other wastes incorporated into building materials.

## EXPERIMENTAL

### Sample collection and preparation

In order to prepare the different building materials, two commercial binders have been used in this study, Ordinary Portland Cement 32.5R (CP) according to EN 197-1^(14)^ and gypsum (Y) according to EN 13279-1^(15)^.

Seven different wastes have been used in this study. The chemical composition of the major components is shown in Table 1.

- Mussel shells (CM) of *Mytilus galloprovincialis* mollusks were collected from an aquaculture industry in the north of Spain. They were composed mainly of CaCO_3_. The material was crushed using a jaw crusher, and the particle size was between 50 and 250 μm. Between 6 000 000 and 8 000 000 tons of shells are produced per year worldwide in the aquaculture industry, and only a quarter of the generated waste is reused^(16)^. In the construction industry, there are previous studies on the use of shell waste instead of limestone in cement production^(17, 18)^ as it requires less energy during the grinding and crushing processes^(19)^.
- Fly ashes (OR), through combustion of residual biomass, were obtained in the olive oil extraction process in a Spanish power plant. The particle size was between 32 and 300 μm. They contained a high potassium level (17.22% wt of K). They might be useful to replace natural gypsum in fire-resistant materials ^(20, 21)^. For each ton of processed olives, ~0.27 tons of olive oil and 0.73 tons of pomace are generated. Only in Andalusia (Spain), 3 000 000 tons per year of pomace are generated. A 30% of the pomace is used for electrical energy, producing more than 50 000 tons of ashes per year^(21)^.
- Fly ashes (COMP) were obtained from the co-combustion of coal (70% wt) and coke (30% wt) in a power plant. Its average size particle was 42 μm. The principal chemical elements were Si and Al, and, to a lesser extent, Fe.
- Fly ashes (LB) were obtained from the combustion of coal (100% wt) in a power plant. Its average size particle was 37 μm. The principal chemical elements were Si and Al. Si content was lower than COMP, but Al content was like COMP. There are several previous studies about the recycling of fly ashes in different building materials (cement, mortars, hydraulic road binders, concrete, bricks and fire-resistance materials)^(22–24)^.
- Bottom ash (ESC) was obtained from the co-combustion of coal (70% wt) and coke (30% wt) in a power plant. Its particle size was between 100 μm and 10 mm. The chemical composition was like that of COMP; they were obtained in the same power plant with the same coal. Bottom ashes were used in concrete as coarse aggregates or were milled and added to cement, bricks or fire-resistant materials^(25–27)^.
- Construction and demolition wastes (RD) were obtained from the ALCOREC plant at San Jose de la Rinconada (Seville, Spain). The plant receives all kinds of mixed waste (concrete, tile, plaster and ceramic), and by a crushing and screening processes, a particle size between 2 and 4 cm was obtained. RD was mainly composed of Si and Ca. Some examples of applications are its use as general bulk fill, sub-base, base or surface material in road construction, hydraulically bound materials or new concrete manufacture^(28, 29)^.
- Blast furnace slag (BFS), a solid waste that was obtained from the iron–steel metallurgical industry was used. Particle size was between 0.1 and 100 μm. The principal chemical elements were Si and Ca. The main use of BFS is in cement production, but it can be used also as any other additive to concrete, hydraulic road binder or as part of alkali-activated materials^(30)^.

**Table 1:** Major elements concentrations of different binders and wastes.

	SiO_2_%	Al_2_O_3_%	Fe_2_O_3_%	MnO %	MgO %	CaO %	Na_2_O %	K_2_O %	TiO_2_ %	P_2_O _5_%	SO_3_ %	Mass loss %
CP	13.8	3.5	2.3	0.1	0.7	59.3	0.1	0.5	0.2	0.1	1.7	15.5
Y	0.1	0.1	0.1	<0.1	<0.1	34.6	0.0	<0.1	<0.1	<0.1	41.7	21.3
CM	<0.1	<0.1	<0.1	<0.1	<0.1	54.0	0.5	<0.1	<0.1	0.1	0.3	43.2
COMP	57.5	23.3	7.1	<0.1	1.8	2.8	0.7	3.8	1.1	0.1	<0.1	1.1
ESC	53.3	25.1	9.2	0.1	1.8	2.4	0.7	3.7	1.5	0.3	0.0	1.1
LB	44.0	24.9	16.1	<0.1	1.4	8.5	0.2	2.9	<0.1	<0.1	0.6	0.7
BFS	35.2	11.6	1.0	<0.1	7.6	43.1	0.2	0.4	<0.1	<0.1	<0.1	<0.1
RD	52.8	9.6	3.7	0.1	2.5	17.9	0.6	1.9	0.5	0.1	0.4	9.2
OR	37.1	7.9	4.0	0.0	6.3	15.3	8.1	9.8	<0.1	1.6	<0.1	9.4

Several binder substitution ratios have been tested in this work using a variety of wastes. The solid components were mixed up in a planetary mixer for 3 min until a homogeneous blend was achieved. Then water was added to the mixture, and it was again mixed during 5 min. After the mixture reached a proper consistency, molds were casted. The densities and compressive strengths of different materials are presented in Table 2. The molds were taken off 24 h later. The samples were cured for 27 d under 25°C and 50% relative humidity. Eleven samples were finally produced for further analysis:

- CP100: It was 100% Portland cement.
- CM80CP20, COMP80CP20, ESC80CP20, LB80CP20 and RD80CP20 were mortars composed by 80% wt by-products and 20% wt of CP. A high proportion of by-products were chosen to analyze the influence of these by-products in the properties of the building materials.
- Y100: It was 100% gypsum.
- Y20OR70V9.5F0.5: It was a fire-resistant panel composed of 20% wt of Y, 70% wt of OR and 9.5% wt of inert vermiculite, and it was a hydrated silicate containing magnesium, aluminum and iron. Additionally, 0.5% polypropylene fiber was added in order to increase the mechanical properties of the final product ^(20)^.
- Y80BFS20, Y60BFS40, Y40BFS60 were gypsum-based mortars incorporating different BFS proportions as indicated in their codes.

**Table 2:** Density and compressive strength of different materials.

Composition	Density (kg per m^3^)	CS (MPa)
CP100	1890 ± 150	41.5 ± 2.5
CM80CP20	1750 ± 130	8.9 ± 0.9
COMP80CP20	1650 ± 125	11.2 ± 1.1
ESC80CP20	1049 ± 102	5.4 ± 0.4
LB80CP20	1500 ± 133	18.3 ± 1.0
RD80CP20	1448 ± 128	21.4 ± 2.1
Y100	1360 ± 95	8.4 ± 0.7
Y80BFS20	1334 ± 90	6.6 ± 0.4
Y60BFS40	1276 ± 83	3.7 ± 0.2
Y40BFS60	1169 ± 78	1.1 ± 0.2
Y20OR70V9.5F0.5	823 ± 55	2.2 ± 0.2

### Gamma-ray spectrometry

Building materials were crushed and then dried at 105°C for 48 h in an oven. Then, they were grounded using a ball mill, sieved, and we collected <2-mm particle size fraction for gamma-ray analyses. The dried samples were put in a polystyrene Petri dish, 80 cm^3^ of capacity, and then they were hermetically sealed for at least 28 d to prevent escape the of radon gas, allowing secular equilibrium of ^226^Ra and ^232^Th with their decay products.

Activity concentration of ^40^K was directly determined through its gamma emission of 1460 keV, while ^232^Th activity concentration was derived through the gamma emissions from ^228^Ac (911 keV). Finally, ^226^Ra activity concentration was determined by means of those from ^214^Pb (351.9 keV).

The main gamma-ray detector was a low-background Canberra high-purity germanium (HPGe) GR-6022 reverse electrode coaxial detector with 60% relative efficiency, surrounded by a 10-cm thick high-purity lead shield. The detector efficiencies were determined using Canberra LabSOCS software (Detector characterization report, ACK # 21217, detector s/n b11555) based on Monte Carlo code in order to take into account the self-absorption corrections for low energy gamma rays and the coincidence-summing effects^(31)^.

Each sample was counted for 48 h, and the activity concentration values were decay-corrected to the sampling date. The spectra were analyzed using Genie 2000 Gamma Analysis Software v3.2. ^(32)^. Uncertainties were reported at 2 sigma (*k* = 2).

The validation of the detector efficiency calibration was based on the successful participation on several ICRM intercomparisons that were related to self-absorption and coincidence-summing corrections^(31, 33)^. Additionally, gamma-ray spectrometry method was verified by analyzing several IAEA reference materials (IAEA-RGU, IAEA-RGTh, IAEA-RGK and IAEA-434 phosphogypsum) and through the successful participation in two ALMERA Proficiency Tests organized by IAEA (IAEA-TEL-2015-01 and IAEA-TEL-2018-04)^(34)^. Finally, Minimum Detectable Activity (MDA) was calculated for ^40^K, ^232^Th and ^226^Ra, obtaining 33 Bq per kg, 8 Bq per kg and 5 Bq per kg, respectively.

## RESULTS AND DISCUSSION

### Activity concentrations

Activity concentration values (Bq per kg) of ^40^K, ^226^Ra and ^232^Th measured in the building materials are shown in Table 3. Most samples had activity concentrations below the average activity concentration for concrete in EU^(12)^ (392 Bq per kg for ^40^K, 60 Bq per kg for ^226^Ra and 35 Bq per kg for ^232^Th); however, some building materials showed higher values for specific radionuclides (Y20OR70V9.5F0.5, COMP80CP20 and ESC80CP20 for ^40^K; Y20BFS80, LB80CP20, COMP80CP20 and ESC80CP20 for ^226^Ra; and LB80CP20, COMP80CP20 and ESC80CP20 for ^232^Th).

**Table 3:** Activity concentration (Bq per kg) and its uncertainty (2 sigma) of natural radionuclides in the analyzed materials.

Sample	^226^Ra (Bq per kg)	^232^Th (Bq per kg)	^40^K (Bq per kg)
CP100	9.3 ± 1.1	7.8 ± 0.8	67 ± 11
CM80CP20	1.8 ± 0.3	N.D.	N.D.
COMP80CP20	78 ± 5	80 ± 4	787 ± 39
ESC80CP20	68 ± 4	72 ± 7	736 ± 49
LB80CP20	181 ± 10	185 ± 10	195 ± 42
RD80CP20	14 ± 2	14 ± 2	238 ± 30
Y100	6.6 ± 0.5	3.5 ± 2.1	67 ± 12
Y80BFS20	30 ± 2	8.7 ± 1.2	75 ± 11
Y60BFS40	56 ± 4	18 ± 2	99 ± 16
Y40BFS60	103 ± 5	33 ± 2	110 ± 20
Y20OR70V9.5F0.5	13 ± 2	13 ± 2	4530 ± 200

N.D. = not detected.

Since Portland cement (CP100) and natural gypsum (Y100) have small activity concentrations (see Table 3), the main contributors to the increase in activity concentration should be the components incorporated into the building material. For COMP80CP20 and ESC80CP20 building materials, the origin of the high radionuclide concentrations of activity is due to the presence of both coal and coke ash, following the same behavior as other trace elements present in these types of raw materials^(35)^. Furthermore, when the fuel used in a power plant changes, although the combustion process is similar, the radiological properties of the ash change greatly, as it can be seen in COMP80CP20 and LB80CP20, though they present similar size distribution and major elements composition.

On the other hand, Y20OR70V9.5F0.5 building material presents a ^40^K activity concentration that is almost one order of magnitude higher than the soil average. Since gypsum has low concentrations of ^40^K as mentioned above (see Table 3), it seems likely that its origin is from the olive stone waste. The olive pomace is naturally enriched during its growth process in potassium (both stable and radioactive isotopes) coming from soil and fertilizers. After its use as a biofuel, olive pomace has K_2_O concentrations up to 9% ^(36)^, which corresponds to ~6800 Bq per kg of ^40^K. If OR ashes are incorporated into a building material in a proportion of 70% w/w, ^40^K activity concentrations can be estimated at around 4800 Bq per kg, which matches with the experimental result for the Y20OR70V9.5F0.5 sample. This high ^40^K activity concentration suggests that the combustion process of olive pomace was carried out at a temperature less than 800–900°C (potassium volatilization point), releasing a large quantity of potassium into the combustion waste.

On the contrary, the CM80CP20 building material did not contain either ^40^K or nuclides from the ^232^Th series. The ^226^Ra (radionuclide from ^238^U series) activity concentration found in this study was the lowest value, which was even lower than that of the Portland cement sample. Potassium concentration in shells was small (<0.1%), as it can be seen in Table 1.

**Figure 1:**
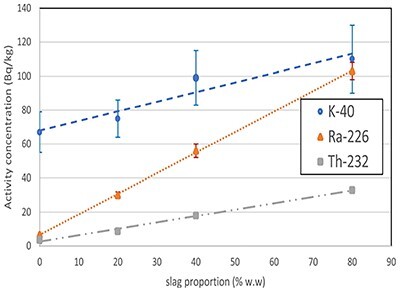
dependence of activity concentrations on the proportion of metal smelters slag mixed with gypsum.

Regarding BFS, activity concentrations of gypsum-based mortars incorporating different BFS proportions are shown in Figure 1. ^40^K activity concentrations were mostly independent of slag contents, considering their uncertainties. However, there was a statistically significant increase of activity concentrations for both ^226^Ra and ^232^Th as the slag content increases. The linear fit to these data showed that there was a good correlation for ^232^Th (*r* = 0.890) and ^226^Ra (*r* = 0.970), allowing the estimation of the activity concentration in pure metals smelter slags (26 and 100 Bq per kg for ^232^Th and ^226^Ra, respectively). These values are in agreement with the previous studies ^(10)^.

Major elements concentrations and the physical properties of the analyzed building materials are shown in Table 1. The results are similar to other results published elsewhere^(27, 37–39)^. A statistically significant positive correlations between the Al_2_O_3_ concentrations and activity concentrations were found for ^226^Ra (*r* = 0.7285; *p* = 0.0110) and ^232^Th (*r* = 0.7884; *p* = 0.0067) and between the Fe_2_O_3_ concentrations and activity concentrations of ^226^Ra (*r* = 0.6108; *p* = 0.0459). This would suggest that, as both compounds are refractory, Ra and Th become more concentrated in these compounds after an incomplete combustion process.

### Absorbed and effective dose rate

To assess the radiological hazard of building materials, gamma radiation dose was evaluated. Activity concentrations of *a*_Ra-226_, *a*_Th-232_ and *a*_K-40_ were transformed in the absorbed gamma dose rate D (nG/h) through conversion factors calculated by the Monte Carlo method^(1)^ at 1 m above the ground (in nGy per h by Bq per kg) as follows:

(1) \begin{equation*} D=0.462{a}_{\mathrm{Ra}-226}+0.604{a}_{\mathrm{Th}-232}+0.417{a}_{\mathrm{K}-40} \end{equation*}

On the other hand, the absorbed dose rate (*D*) was converted into the annual effective dose indoors (*E* (mSv/y)) by applying equation (2). The conversion value *Q* was 0.7 Sv per Gy for environmental exposure to gamma rays; time *T* was 1 y (8760 h); and 0.8 was the indoor occupancy factor indicating that 80% of time was spent indoors, as recommended by UNSCEAR (1998) ^(40)^.

(2) \begin{equation*} E=T\cdot Q\cdot D\cdot 0.8\cdot{10}^{-6} \end{equation*}

The maximum estimated values of the absorbed dose rate and the annual effective dose for all samples are presented in Table 4. The absorbed dose rate of most samples was lower than the world average indoor absorbed dose rate (84 nGy per h) as reported in UNSCEAR (Annex B)^(1)^. Regarding the maximum annual effective dose, all the values are below the reference value (1 mSv per y) for public exposure^(11)^. Two samples (LB80CP20 and Y20OR70V9.5FO.5) are presenting values nearby the reference value.

**Table 4:** Activity concentration indexes, radium equivalent activity, absorbed and effective dose rate for analyzed samples.

Sample	*I*	*I_d_*	Ra_eq_ (Bq per kg)	*D* (nGy per h by Bq per kg)	*E* (mSv per y)
CP100	0.092 ± 0.007	0.07 ± 0.03	25.6	11.8	0.058
CM80CP20	0.006 ± 0.001	0.004 ± 0.004	14.2	6.5	0.032
COMP80CP20	0.92 ± 0.03	0.65 ± 0.10	253.0	117.2	0.575
ESC80CP20	0.83 ± 0.04	0.5 ± 0.1	227.6	105.6	0.518
LB80CP20	1.59 ± 0.06	0.7 ± 0.1	460.6	203.5	0.998
RD80CP20	0.20 ± 0.02	0.12 ± 0.05	52.3	24.8	0.122
Y100	0.06 ± 0.01	0.04 ± 0.01	16.8	8.0	0.039
Y80BFS20	0.17 ± 0.01	0.09 ± 0.03	48.2	22.2	0.109
Y60BFS40	0.31 ± 0.02	0.17 ± 0.05	89.4	40.9	0.201
Y40BFS60	0.55 ± 0.02	0.27 ± 0.05	158.7	72.1	0.354
Y20OR70V9.5F0.5	1.62 ± 0.07	0.6 ± 0.1	380.4	202.8	0.995

### Activity concentration index

Additionally, following the recommendations established by the European Commission Radiation Protection 112 technical guide^(41)^, we have applied the activity concentration index *I* as a tool for building materials screening despite the fact the limitations of such a tool are well established^(12)^. The activity concentration index defined according to^(1)^ is given by:

(3) \begin{equation*} I=\frac{a_{\mathrm{Ra}-226}}{300}+\frac{a_{\mathrm{Th}-232}}{200}+\frac{a_{K-40}}{3000} \end{equation*}

*a_x_* being the activity concentration (Bq per kg) of the nuclide *x* in the building material evaluated.

*I* is a tool that is widely used to assess the gamma radiation dose, in excess of typical outdoor exposure, that an individual may receive from the building materials. However, *I* does not take into account the density and thickness of the construction material being calculated using the concrete density (2350 kg per m^3^) and 0.2 m of thickness^(10)^. Therefore, *I* has to be modified with two weighting factors related to density and thickness^(42)^ according to the equation:

(4) \begin{equation*} {I}_d=\left(\frac{a_{\mathrm{Ra}-226}}{300}+\frac{a_{\mathrm{Th}-232}}{200}+\frac{a_{\mathrm{K}-40}}{3000}\right)\cdotp \frac{\rho \mathrm{th}}{470} \end{equation*}

where *ρ* is the density of the material (kg per m^3^), th is the thickness (m) and 470 is the weight per unit area (kg per m^2^) according to the model of the European radiological protection principles^(43)^.

Both activity concentration indexes of these materials are summarized in Table 4. Despite that it is not quite usual in the literature, uncertainties of the index *I* were calculated according to GUM^(44)^ in order to test the statistical significance of the results. The recommendations from the European Commission^(43)^ for bulk materials are that the effective dose rate reaches up to 0.3 mSv per y if *I* index was ≤ 0.5 (or if *I* ≤ 1.0, the effective dose rate reaches up to 1 mSv per y). For superficial and other materials with restricted use, if *I* ≤ 2, excess effective dose rate reaches up to 0.3 mSv per y, or if *I* ≤ 6, the excess effective dose rate reaches up to 1 mSv per y.

As shown in Table 4, 5 out of 11 samples produced a low *I* index: Portland cement; mixes of Portland cement with mussel shells and construction and demolition wastes; pure gypsum and a mix of 80% gypsum and 20% BFS. The sample CM80CP20 produced the lowest dose and *I* index, being even six and four times less than those of cement and gypsum, respectively. This finding suggests the possibility to use this matrix as a dilution agent for more troublesome materials. The composite material containing 40% of BFS also produces an effective dose rate below 0.3 mSv per y. When the proportion of slag raises to 80%, the effective dose rate exceeds 0.3 mSv per y, as for samples ESC80CP20 and COMP80CP20; and their use in bulk amounts should be restricted, although they could be used as superficial materials or for restricted use (tiles, boards, etc.). Finally, the samples containing fly ashes from coke and coal co-combustion and olive stones produced high values of *I* index (1.6 for both) and subsequently increments on the effective dose rate close to 1 mSv per y.

Table 4 shows the *I_d_* for the different compositions using the real density of the construction materials (Table 2) and assuming a thickness of 0.2 m. When the density is considered, the activity concentration index decreased in all cases because its density is lower than concrete, but, additionally, this decrease is higher in materials containing biomass ashes.

Under these conditions, it can be concluded that the mixing of mussel shells could allow that certain by-products could be recycled as construction materials after mixing; otherwise, their radiological characteristics could restrict their use as construction materials. This fact seems to open a new way of using this by-product on an industrial scale as a cheap and reliable complement to some other materials that, despite their good performances in some applications as construction materials, could find troublesome application due to the radiological and regulations implications of their use.

### Radium equivalent activity

Radiological health hazards of ^226^Ra, ^232^Th and ^40^K non-uniformity distribution in building materials could also be estimated by using an index, the radium equivalent activity (Ra_eq_). This index is a weighted sum of the activity concentrations of the three radionuclides, assuming that 370 Bq per kg of ^226^Ra, 259 Bq per kg of ^232^Th or 4810 Bq per kg of ^40^K produce the same gamma dose due to the external gamma dose. It is defined as follows^(45)^:

(5) \begin{equation*} {\mathrm{Ra}}_{\mathrm{eq}}={a}_{\mathrm{Ra}-226}+1.43{a}_{\mathrm{Th}-232}+0.077{a}_{\mathrm{K}-40} \end{equation*}

*a_x_* being the activity concentration (Bq per kg) of the nuclide *x* in the building material evaluated.

The maximum value of Ra_eq_ must be lower than 370 Bq per kg for a radioactive-safe use of the building materials, keeping the external dose below 1.5 mGy per y.

The Ra_eq_ of the samples are presented in Table 4. It can be noticed that only two samples, those containing fly ashes from the coke and coal co-combustion and olive stones, present radium equivalent activity values that are higher than the recommended value (370 Bq per kg). The other samples can be considered within the acceptable limits. The lowest values were obtained for Portland cement; mixture of Portland cement with mussel shells and construction and demolition wastes; pure gypsum and a mix of 80% gypsum and 20% BFS. The sample CM80CP20 produced the lowest value (14.2 Bq per kg). The composite materials containing BFS also produce Ra_eq_ values lower than 370 Bq per kg (with a maximum of 158.7 Bq per kg for 80% BSF composition). Higher values were determined for samples ESC80CP20 and COMP80CP20, being 227.6 and 253 Bq per kg, respectively, but lower than the recommended value.

## CONCLUSIONS

The radiological impact of the potential use of novel wastes as construction materials has been analyzed by assessing the effective dose rate, radium equivalent activity and activity concentration index. The results show that high concentrations of certain natural radionuclides in several of these matrices lead to exceeding of the recommendations established by the European Commission. Such high activity concentrations seem to be related to the origin of the samples, especially those coming from the combustion or co-combustion of oil coke and coal and a matrix containing olive pomace ashes enriched in natural potassium during its low temperature combustion. On the contrary, a material based in mussel shells showed very low activity concentrations, leading to low effective dose rate and activity concentration index. The results show that several of the analyzed matrices exceed the EC limits, but they could be used after mixing with almost pure matrices, such as cement, gypsum and, especially, mussel shells. In this way, new applications of this material appear as a cheap and reliable dilution agent, allowing the use of some other by-wastes as construction materials within the EC limits.

## CONFLICT OF INTEREST

No potential conflict of interest was reported by the author(s).
